# Decision tree models to guide endotracheal tube selection in dogs

**DOI:** 10.3389/fvets.2026.1894775

**Published:** 2026-10-21

**Authors:** Nuttawadee Ngamlertwong, Veerasak Punyapornwithaya, Supitchaya Siriyakhun, Chris Seymour, Seri Seki

**Affiliations:** 1 Faculty of Veterinary Medicine, Chiang Mai University, Chiang Mai, Thailand; 2 Research Center for Veterinary Biosciences and Veterinary Public Health, Chiang Mai, Thailand; 3 Retired, Ludlow, United Kingdom; 4 School of Veterinary Nursing and Technology, Faculty of Veterinary Science, Nippon Veterinary and Life Science University, Musashino, Japan

**Keywords:** anatomical parameters, canine, decision tree, endotracheal tube, machine learning

## Abstract

**Introduction:**

Choosing an endotracheal tube (ETT) of an appropriate size is critical for effective airway management in dogs undergoing anesthesia or emergency care. Conventional methods often rely on body weight (BW), palpation of external tracheal diameter, nasal septum width, or subjective clinician experience. However, these approaches are frequently imprecise and lack well-established guidelines. Therefore, this study aimed to develop a machine learning-based decision tree model to standardize ETT size using measurable anatomical parameters in dolichocephalic, mesocephalic, and brachycephalic breeds.

**Methods:**

A retrospective analysis was conducted on data from dogs undergoing anesthesia or emergency care at the Northern Regional Veterinary Healthcare and Learning Center, Faculty of Veterinary Medicine, Chiang Mai University, (NOVEL CMU) Thailand between January and December 2024. We investigated the relationship between the internal diameter (ID) of the ETT used and multiple patient variables, including BW, sex, age, breed, and internal tracheal diameter (ITD) measured at the thoracic inlet on radiographs. Decision tree analysis was used to determine the ETT selection. We hypothesized that a decision tree model integrating BW and ITD would accurately predict appropriate ETT sizing in dogs, but that upper airway anatomical differences would necessitate separate models for non-brachycephalic breeds (Group DM, including dolichocephalic and mesocephalic conformation) and brachycephalic breeds (Group B).

**Results and discussion:**

The results demonstrated that Group B required ETTs with a significantly smaller ID compared to Group DM of the same BW. Variable importance analysis identified BW and ITD as the primary predictors of ETT size in both groups. Conversely, sex and age demonstrated negligible predictive value and were not significant factors in the selection process. Hence, the decision tree model successfully generated distinct algorithmic pathways for Group DM and Group B. The developed decision tree models offer a practical, non-invasive method for standardizing ETT sizes in clinical practice, thereby enhancing skilled veterinary human capital. This algorithmic approach accounts for individual anatomical variations in the respiratory tract, particularly in brachycephalic dogs. For clinical application, clinicians should prepare tubes within ±0.5 mm of the predicted size to ensure optimal airway management and patient safety.

## 1 Introduction

Endotracheal intubation secures the airway during anesthesia, reducing the risk of regurgitation-induced aspiration pneumonia while facilitating oxygenation and volatile anesthetic delivery (1–3). Selecting an appropriately sized endotracheal tube (ETT) also helps to prevent the release of waste anesthetic gases (WAGs) into the environment (4) and minimizes exposure of healthcare personnel, which could pose a significant health risk (5–7). However, choosing an appropriately sized ETT can be challenging for inexperienced clinicians. Ideally, the largest ETT that can be passed atraumatically through the arytenoid cartilages should be selected.

Formulas have been established to estimate correct ETT size in children based on phenotypic parameters such as body weight (BW) and age. In dogs, however, predictive models are problematic due to breed-related variability in tracheal anatomy. Common methods to estimate ETT size have used BW (8), external palpation of the trachea, and nasal septal width. In dolichocephalic breeds, the percentage of the best-fit tube selection using either nasal septal width or external tracheal palpation was 21, and 46%, respectively (9). Phenotypic traits, including BW and the vertical length of the fourth digital pad exhibit linear correlations with uncuffed polyvinyl chloride ETT size in Dalmatian puppies (10). These data show that there are several methods to predict appropriate ETT size by measuring aspects of morphology in specific breeds. Predicting the appropriate ETT size in brachycephalic dogs is particularly difficult because a narrow cricoid cartilage (11) and tracheal hypoplasia (12, 13) restricts airway dimensions and causes weight-based predictions to fail.

Internal tracheal diameter (ITD) has also been used to predict an appropriate ETT size. Thoracic radiography may be undertaken to assess cardiopulmonary health prior to anesthesia (14) but also permits measurement of ITD at the thoracic inlet. A study in Beagle dogs showed that the optimal ETT outer diameter (OD) was equal to 70% of the ITD measured via thoracic radiography (15).

Our study employs decision tree analysis to determine the relative importance of selected variables to predict ETT size in several dog breeds. Decision tree models have been previously applied to classify wildlife species from imagery (16), optimize livestock yield and welfare (17, 18) and detect diseases in companion animals (19, 20).

The objectives of this study were twofold: first, to investigate the relationship between the ETT and patient-specific variables, including gender, age, BW, and ITD for non-brachycephalic breeds (Group DM, including dolichocephalic and mesocephalic conformation) and brachycephalic breeds (Group B); and second, to develop a predictive model using a decision tree algorithm to reproduce selection of ETTs based on clinical experience, providing a valuable clinical decision support system for novice anesthetists. We hypothesized that a decision tree model incorporating BW and ITD could effectively predict ETT size in dogs, and that separate predictive models would be required for Group DM and Group B owing to upper airway anatomical differences.

## 2 Materials and methods

### 2.1 Study design and setting

This study was designed as a retrospective, observational, hospital-based investigation. Medical records and thoracic radiographs of dogs that had undergone clinical procedures requiring endotracheal intubation between January 1 and December 31, 2024 at the Northern Regional Veterinary Healthcare and Learning Center, Faculty of Veterinary Medicine, Chiang Mai University, (NOVEL CMU) Thailand.

### 2.2 Study population

All patients that underwent clinical procedures requiring endotracheal intubation during the study period were screened using the hospital's electronic medical record system. Inclusion criteria were dogs requiring anesthesia for diagnostic procedures such as computed tomography (CT scans) or surgery, and which had thoracic radiographs available prior to anesthesia. Exclusion criteria were cases recorded as duplicated entries; animals of other species (e.g., feline or rabbit); cases without available pre-anesthetic thoracic radiographs; and patients whose thoracic radiographs were obtained as part of an investigation of severe disease requiring medical treatment.

### 2.3 Data collection

Details of breed, sex, age, BW, and the internal diameter (ID) of the ETT used during anesthesia were recorded. All endotracheal intubations were performed using cuffed tubes sourced from two manufacturers based on tube size availability at the time of the procedure: polyurethane tubes (Well Lead Medical Co., Ltd.) and polyvinyl chloride tubes (Zhanjiang Star Enterprise Co., Ltd.). The ITD (mm) at the thoracic inlet, defined as the region immediately cranial to the first rib (21), was measured on lateral thoracic radiographs. For each dog, the ITD was measured three times by the same investigator to minimize random measurement error, and the mean of the three measurements was used for all subsequent analyses.

### 2.4 Data stratification and sampling

The study population was stratified *a priori* into two anatomically distinct groups based on established breed classifications and veterinary literature (22, 23): Group DM and Group B. Borderline or variable breeds, such as the Chihuahua, were assigned to Group B based on veterinary literature and their documented risk profile for upper airway anatomical variations (23). This stratification was performed to account for structural airway differences that might systematically influence the ETT used. Within each group, the dataset was randomly partitioned into training (70%) and testing (30%) subsets using functions implemented in the caret package (24) in R (version 4.4.2). The complete R script for this data partitioning is available in the Supplementary Data. A fixed random seed was applied to ensure reproducibility. The 70:30 allocation was selected to provide sufficient observations for model estimation while preserving an independent dataset for external performance evaluation.

For the ITD measurement, to quantify within-dog measurement variability, the standard deviation (SD) of the three repeated measurements was first calculated for each dog. The mean of these individual SD values was then calculated across all dogs and reported as the mean within-dog SD, representing the average measurement variability within individual dogs.

In addition, to assess the repeatability of the measurements, the intraclass correlation coefficient (ICC) was calculated using a two-way mixed-effects model with absolute agreement based on the three repeated measurements obtained from each dog. Both the ICC for a single measurement and the ICC for the average of three measurements were estimated, together with their 95% confidence intervals (CIs). The single-measurement ICC represents the reliability of an individual measurement, whereas the average-measurement ICC reflects the reliability achieved by averaging three repeated measurements. Statistical significance was assessed using the corresponding *F*-test, with *P* < 0.05 considered statistically significant.

### 2.5 Decision tree

The predictor variables included: BW (kilograms, kg); ITD (millimeters, mm); age (months); and sex. BW, ITD and age are continuous variables, whilst sex is a categorical variable. The outcome variable was ETT size, defined as the ID of ETT (mm) selected for intubation. Continuous predictors were retained on their original measurement scales to preserve clinical interpretability. The algorithm constructs a binary decision tree by recursively partitioning the predictor space into increasingly homogeneous subsets with respect to the outcome.

During tree construction, split selection at each internal node was determined by minimizing the residual sum of squares (RSS), defined as:


$$\begin{array}{l} RSS=\sum_{i=1}^{n}(y_{i}-{\hat{y}}_{i}{)}^{2} \end{array}$$

where *y*_*i*_ denotes the observed ETT size and ŷ_*i*_ represents the predicted value within a given node. For each candidate split, the reduction in RSS relative to the parent node was calculated, and the split yielding the greatest decrease in RSS was selected. This recursive process continued until predefined stopping criteria were met, after which cost-complexity pruning was applied to obtain the final model.

### 2.6 Cross-validation and hyperparameter optimization

Model training incorporated repeated k-fold cross-validation to optimize hyperparameters and improve generalizability. Specifically, 10-fold cross-validation repeated 10 times was applied to the training dataset. During each fold, the dataset was partitioned into 10 approximately equal subsets; nine subsets were used for model fitting and one subset for validation. This process was repeated across folds and iterations, yielding 100 resampling procedures (25).

The optimal complexity parameter was selected based on the minimum cross-validated prediction error. When applicable, the one-standard-error rule was used to select a more parsimonious model within one standard error of the minimum error estimate, thereby enhancing model stability (25).

A regression decision tree was developed to predict the ETT using the rpart package in R (version 4.4.2). The model was fitted to the training dataset using the analysis of variance (ANOVA) splitting method. Ten-fold cross-validation was performed to evaluate the tree complexity. The initial complexity parameter was set at 0.001. A node was eligible for splitting when it contained at least 15 observations, while each terminal node was required to contain at least seven observations. The maximum tree depth was limited to 30 levels.

### 2.7 Model performance evaluation

Predictive performance was evaluated using both internal validation within the training dataset and independent validation using the testing dataset. Performance assessment focused on quantifying the magnitude and stability of prediction error to determine model accuracy and generalizability.

Model accuracy was primarily evaluated using root mean square error (RMSE), which measures the average magnitude of prediction error while assigning greater weight to larger deviations. In addition, mean absolute scaled error (MASE) was calculated as a scale-independent metric of predictive accuracy. MASE facilitates comparison across groups by standardizing prediction error relative to a naïve benchmark model. A MASE value below 1 indicates that the decision tree performs better than the reference naïve approach, whereas values above 1 suggest inferior performance.

Model generalizability was assessed by comparing predictive performance between the training and testing datasets within each group. After model development using repeated cross-validation on the training data, the finalized regression tree was applied to the independent testing dataset. Performance metrics obtained from both datasets were systematically compared to evaluate model stability, potential overfitting, and external validity. Consistency of predictive accuracy between training and testing results was interpreted as evidence of robust generalization, whereas substantial discrepancies were considered indicative of reduced model stability.

### 2.8 Variable importance assessment

Assessment of variable importance provides insight into the relative contribution of each predictor to model performance and helps identify the most influential clinical factors associated with ETT size selection (26). Variable importance was quantified using the *varImp* function within the caret framework (24). The resulting importance values were scaled to facilitate comparison among predictors. Higher importance scores indicate a greater relative contribution of the variable to reducing prediction error and to the overall predictive structure of the model.

### 2.9 Model interpretation

The final pruned decision tree was graphically visualized to display the hierarchical decision structure. Internal nodes represent optimal binary splits based on predictor thresholds, while terminal nodes represent predicted ETT sizes. The tree structure allows direct clinical interpretability by identifying dominant predictors and clinically relevant cut-off values influencing ETT size selection.

### 2.10 Ethical approval

Ethical approval was obtained from the Institutional Review Board (IRB) prior to data analysis. This retrospective review was deemed exempt from full review as it involved retrospective animal-records design. The assigned exempt approval number for this study is EX68005, from the Chiang Mai University institutional animal care and use committee (FVM – ACUC).

## 3 Results

### 3.1 Data

Medical records were reviewed for 1,448 animals that underwent endotracheal intubation during 2024. Of these, 868 cases were excluded for the following reasons: duplicate data (*n* = 39), erroneous data (*n* = 6), absence of thoracic radiographs (*n* = 359), non-canine species including cats (*n* = 410) and a rabbit (*n* = 1), and animals that had received medical therapy and thoracic radiography due to illness (*n* = 53). Data from the remaining 580 canines met the inclusion criteria and were analyzed in this study, as shown in Figure 1.

**Figure 1:**
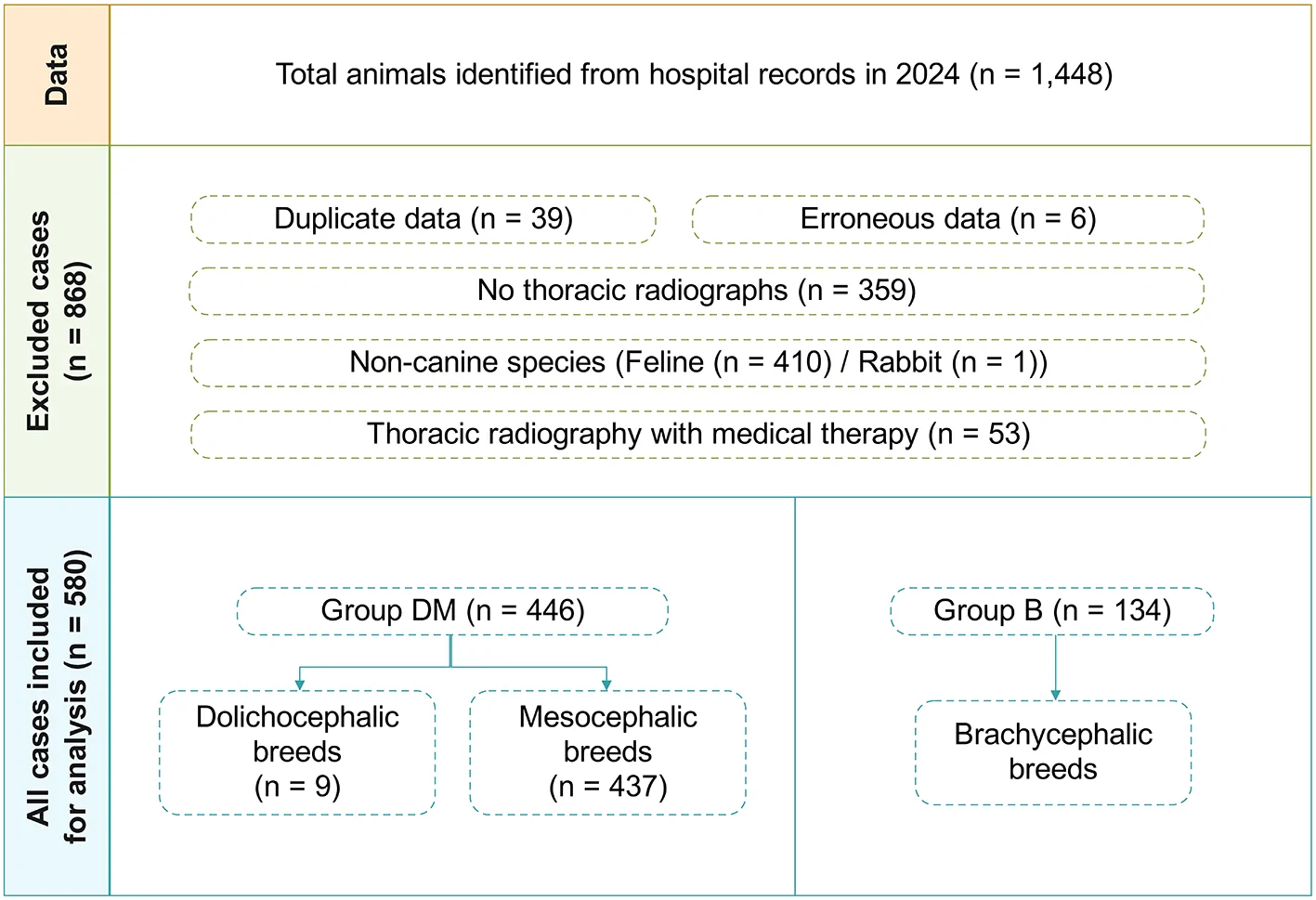
Flowchart of case screening, exclusion criteria, and classification. Overview of the selection pipeline for animals identified from NOVEL CMU in 2024. A total of 868 cases were excluded due to duplicate entries, erroneous data, lack of thoracic radiographs, non-canine species (felines and rabbits), or thoracic radiography performed alongside medical therapy. The final included canine cohort (*n* = 580) was stratified by skull conformation into the dolichocephalic and mesocephalic dogs (Group DM, *n* = 446) and the brachycephalic dogs (Group B, *n* = 134) for decision-tree predictive modeling of endotracheal tube sizing.

### 3.2 Descriptive data

In Group DM, a total dataset of 446 animals was included in the analysis. Of these, 214 (47.99%) were male and 232 (52.01%) were female. A wide range of breeds in Group DM were represented: Alaskan Malamute, American Cocker Spaniel, American Pitbull, American Bully, Australian Shepherd, Beagle, Bangkaew, Borzoi, Bull Terrier, Corgi, Dachshund, Dalmatian, Doberman Pinscher, German Shepherd, German Shorthaired Pointer, Golden Retriever, Greyhound, Great Dane, Italian Gray hound, Jack Russell Terrier, Labrador Retriever, Miniature Pinscher, Miniature Schnauzer, Saint Bernard, Siberian Husky, Spitz, Terrier, Thai Ridgeback, West Highland White Terrier, Wire-Haired Fox Terrier, Papillon, Pomeranian, Poodle, Rottweiler, Shiba Inu, Yorkshire Terrier and mixed breed. In Group B, a total dataset of 134 animals was included in the analysis. Of these, 60 (44.78%) were male and 74 (55.22%) were female. Dog breeds included: English Bulldog, Chinese Shar-pei, French Bulldog, Pekingese, Pug, Shih Tzu, Chihuahua, and Chow Chow.

Figure 2 presents the data distributions for age, BW, and ITD for Groups DM and B; Supplementary Figure 1 illustrates the frequency distribution of the utilized ETTs for both groups. An analysis of these distributions reveals that age is comparable between the two groups. In contrast, Group B exhibits a lower BW than Group DM, alongside a substantially smaller weight range. Furthermore, the ITD is narrower in Group B than in Group DM.

**Figure 2:**
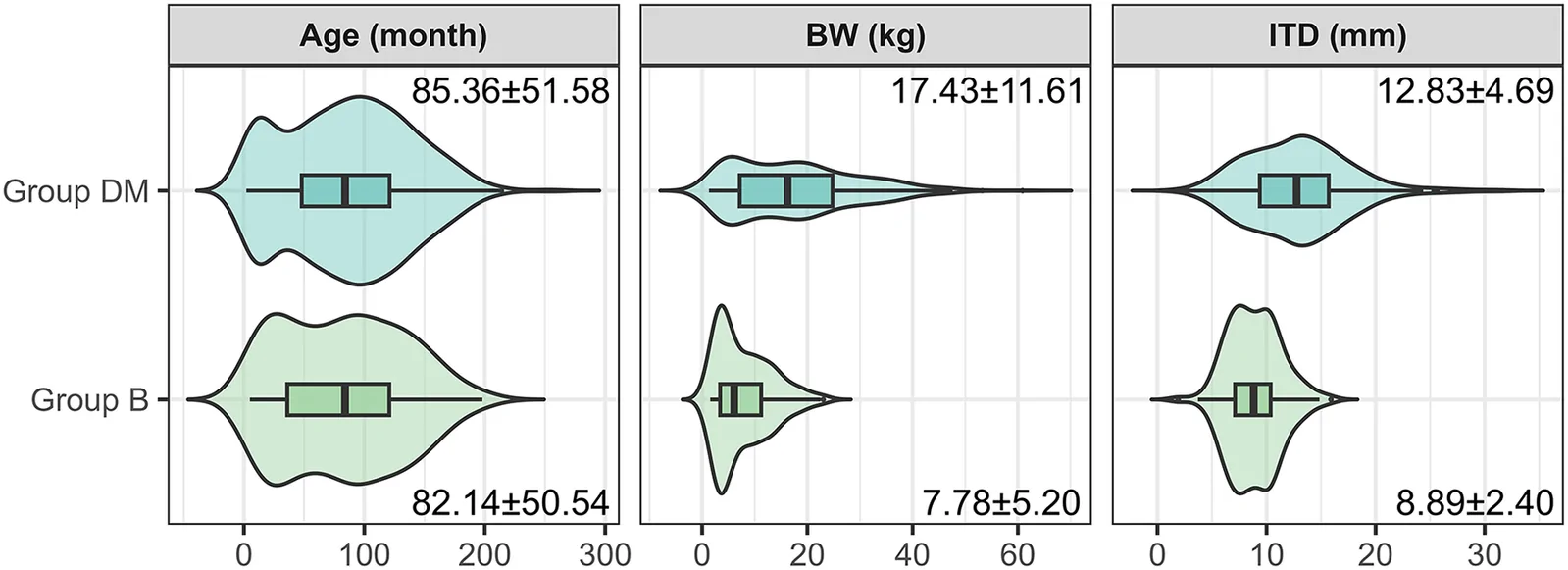
Descriptive statistics and distribution of key anatomical factors, stratified by dolichocephalic and mesocephalic dogs (Group DM) and brachycephalic dogs (Group B). Violin plots depict the data distribution and probability density of clinical parameters including age (months), body weight [BW; kilograms (kg)], and internal tracheal diameter [ITD; millimeters (mm)]. Within each plot, the internal box plot displays the median, interquartile range (IQR), while numerical annotations indicate the mean and standard deviation (SD).

The three repeated ITD measurements demonstrated minimal within-dog variability, with a mean within-dog standard deviation of 0.1899 mm. Consistent with this finding, the intraclass correlation coefficient (ICC) indicated excellent repeatability, with a single-measurement ICC of 0.997 (95% CI: 0.996–0.998, *P* < 0.001) and an average-measurement ICC of 0.999 (95% CI: 0.998–1.000, *P* < 0.001). These results indicate that variation among repeated measurements obtained from the same dog was minimal, whereas the observed variability was primarily attributable to true differences between dogs rather than measurement error. Furthermore, the slightly higher ICC for the average of three measurements suggests that averaging repeated measurements further reduces random measurement error and provides a more precise estimate than a single measurement.

### 3.3 Variable importance

In both Group DM and Group B, important factor variables demonstrated a highly significant association between actual ETT and both BW and ITD. Both BW and ITD were the dominant predictors of ETT across both groups, although their relative contributions varied. Specifically, BW and ITD accounted for 80 and 57.9% of the predictive importance in Group DM, compared to 68.7 and 36.7% in Group B. In contrast, sex and age contributed minimally to the model, exhibiting variable importance scores of 0.7% for age and 2.3% for sex in Group DM, compared to 12.8% for age and 0.7% for sex in Group B as shown in Figure 3.

**Figure 3:**
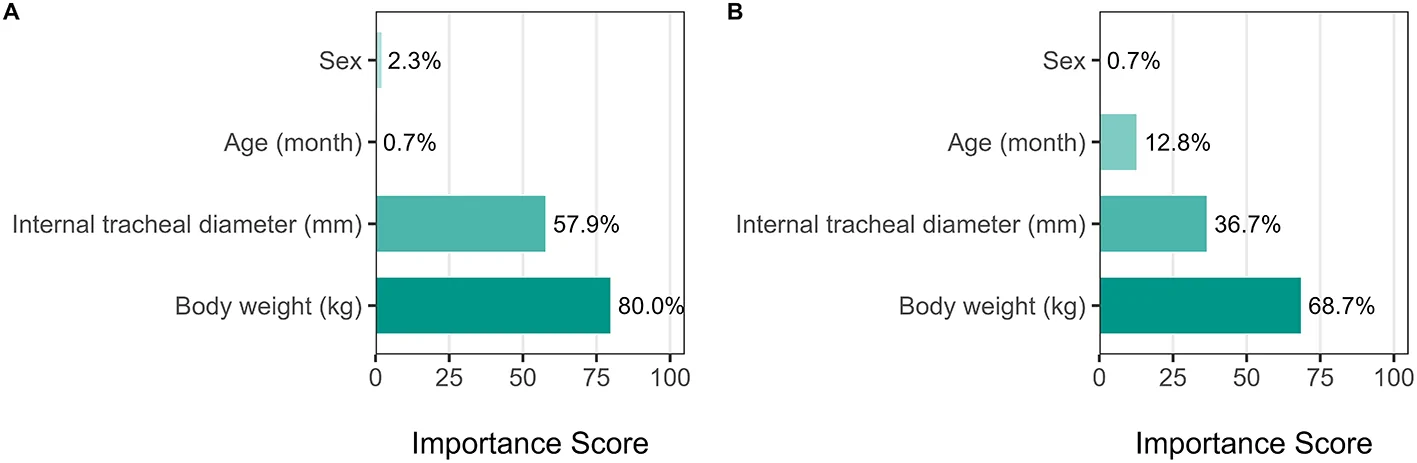
Variable importance analysis illustrates the relative influence of predictor variables on endotracheal tube selection for **(A)** dolichocephalic and mesocephalic dogs (Group DM) and **(B)** brachycephalic dogs (Group B). Bar charts show the relative importance scores (%) of sex, age (months), internal tracheal diameter [ITD; millimeters (mm)], and body weight [BW; kilograms (kg)].

Furthermore, the decision tree and random forest models produced a consistent ranking of the two most important predictors. BW was identified as the most influential predictor of ETT size, followed by ITD, in both modeling approaches (Supplementary Figure 2). This agreement indicates that the importance of these variables was robust to the choice of modeling method and was not solely dependent on the structure of a single decision tree. The consistency between the two models strengthens the evidence that BW and ITD are the principal predictors of ETT size in this dataset.

### 3.4 Prediction of appropriate ETT by using the decision tree

Decision tree analysis (Figures 4, 5) establishes a clinical decision diagram for ETT selection based on the hierarchical importance of the identified predictors. The ETT decision tree of Group DM (Figure 4) initiates the primary split at a BW of 10.4 kg, partitioning the group into lower-weight (31.4%, *n* = 98) and higher-weight (68.6%, *n* = 214) branches. This hierarchical structure provides an uncomplicated method for tube selection. For patients weighing less than 4.68 kg, the model indicates a 3.8 mm ID of ETT. Given that clinical ETT sizes are standardized in 0.5 mm increments (e.g., 3.0, 3.5, 4.0), the calculated 3.8 mm value is rounded up to a 4.0 mm ID tube. The clinical objective is to select the largest possible ETT that avoids respiratory tract trauma, which optimizes ventilation by reducing airway resistance while maintaining mucosal integrity. To achieve this, ETT selection was always rounded up toward the largest appropriate diameter to optimize visualization and access through the respiratory tract. To reconcile this strategy and ensure a proper fit despite anatomical variations, backup ETTs sized ID relative to the primary tube were prepared prior to intubation. Corresponding ID and OD values of ETTs are illustrated in Supplementary Figures 3, 4. However, for the intermediate BW group (BW 4.68–10.4 kg), the decision is jointly determined by BW and ITD. In this range, ITD acts as a critical secondary determinant: an ITD threshold of < 7.77 mm predicts a 4.5 mm tube, whereas an ITD ≥ 7.77 mm predicts a 5.5 mm tube. For patients over 10.4 kg, the decision pathway is simpler, with BW serving as the exclusive predictor. The model stratifies these patients into sequential weight classes with corresponding tube sizes: 10.4–15.5 kg (6.5 mm), 15.5–20 kg (7.5 mm), 20–25.9 kg (8.0 mm), and 25.9–33 kg (8.5 mm). While a 9.5 mm ETT is indicated for patients exceeding 33 kg, its clinical application is constrained by the maximum 10.0 mm tube size available in the NOVEL CMU.

**Figure 4:**
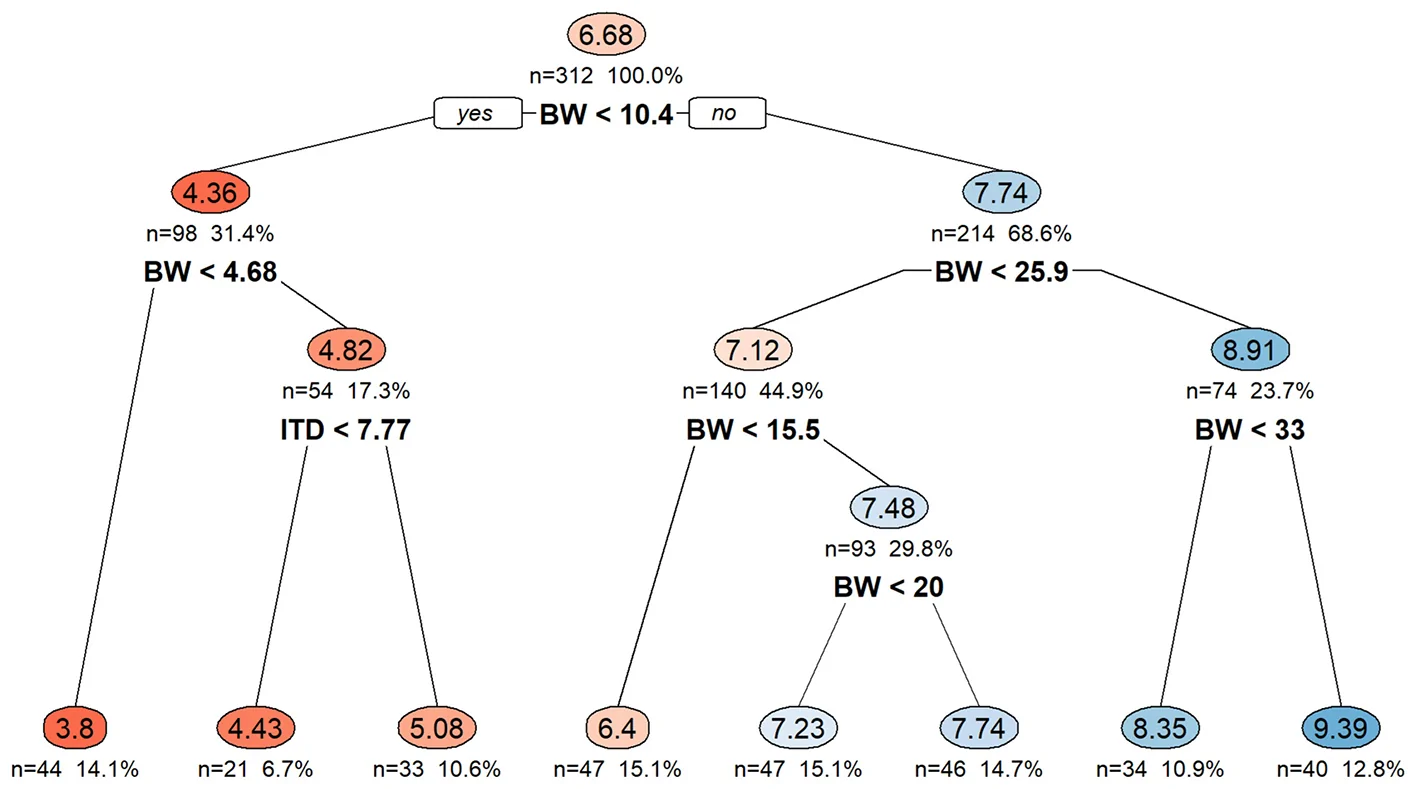
Decision-tree regression model for selecting endotracheal tube (ETT) size in dolichocephalic and mesocephalic dogs (Group DM). The diagram illustrates the decision tree split hierarchy based on body weight [BW; kilograms (kg)] and internal tracheal diameter [ITD; millimeters (mm)] for dolichocephalic and mesocephalic dogs. Decision rules at each node split the data into left (yes) and right (no) pathways based on specific threshold conditions (e.g., BW < 10.4 kg or ITD < 7.77 mm). Oval nodes display the mean predicted ETT size, alongside the sample size (*n*) and percentage of the training study contained within that node. The bottom terminal nodes display the final predicted ETT size for each subgroup.

**Figure 5:**
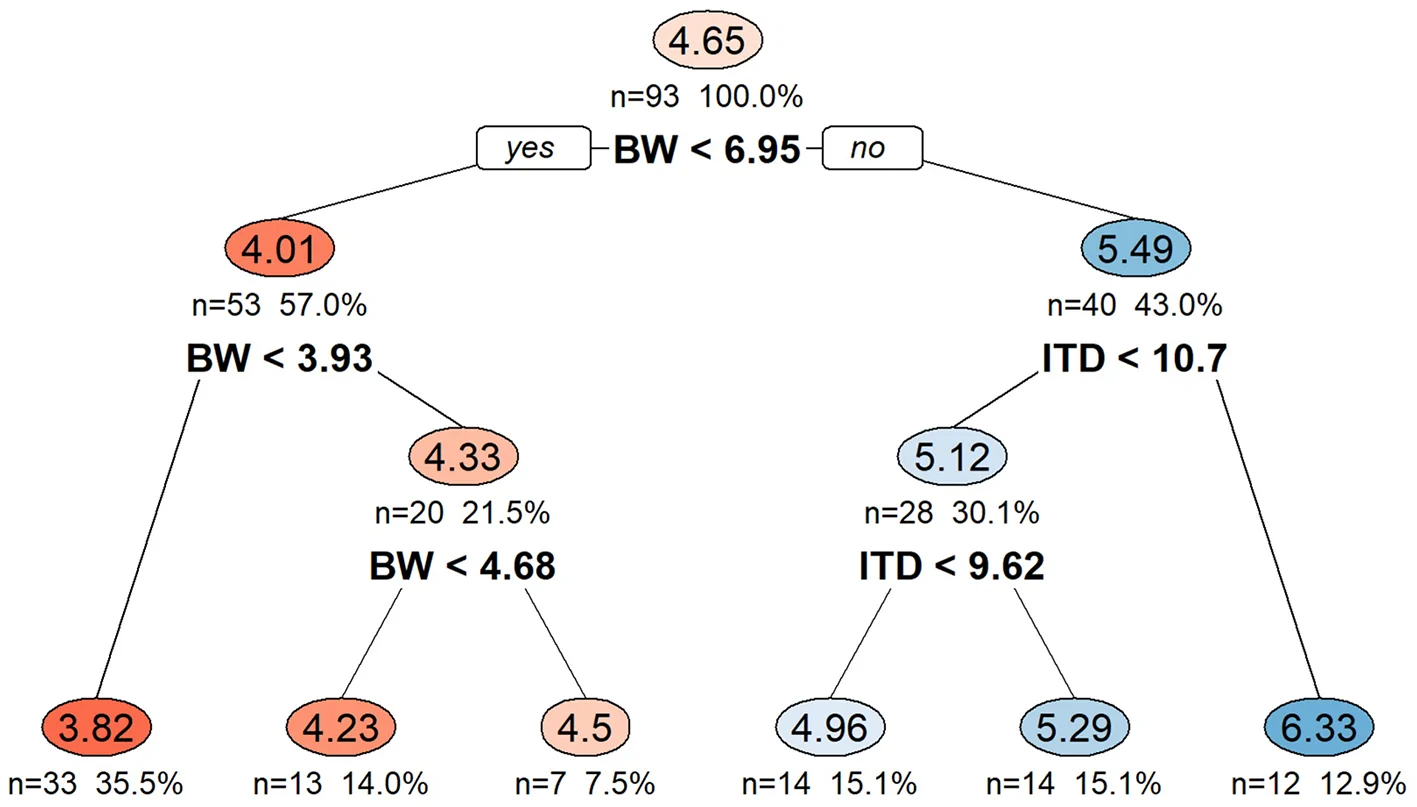
Decision-tree regression model for selecting endotracheal tube (ETT) size in brachycephalic dogs (Group B). The diagram illustrates the decision tree split hierarchy based on body weight [BW; kilograms (kg)] and internal tracheal diameter [ITD; millimeters (mm)] for brachycephalic dogs. Decision rules at each node split the data into left (yes) and right (no) pathways based on specific threshold conditions (e.g., BW < 6.95 kg or ITD < 10.7 mm). Oval nodes display the mean predicted ETT size, alongside the sample size (*n*) and percentage of the training study contained within that node. The bottom terminal nodes display the final predicted ETT size for each subgroup.

In contrast, the decision tree of Group B (Figure 5) establishes a straightforward algorithmic method for tube selection, starting with a primary split at a BW of 6.95 kg. This threshold partitions the group into lower-weight (< 6.95 kg; 57%, *n* = 53) and higher-weight (≥6.95 kg; 43%, *n* = 40) branches. For patients weighing less than 6.95 kg, the decision is based solely on weight: those < 3.93 kg require a 4.0 mm tube, those 3.93–6.95 kg require 4.5 mm tube. Conversely, for patients exceeding 6.95 kg, the model incorporates ITD to ensure accuracy. Specifically, an ITD < 9.62 mm indicates a 5.0 mm tube, while an ITD of 9.62–10.7 mm indicates a 5.5 mm tube. Finally, for patients with an ITD exceeding 10.7 mm, a 6.5 mm tube is suggested.

To evaluate safety, predicted ETT OD was compared relative to ITD across groups. Across all animals, 91.0% (528/580) had a predicted OD ≤ ITD, suggesting a minimal likelihood of airway injury. This safety margin was consistently observed in both Group DM (91.9%, 410/446) and Group B (88.1%, 118/134). Potential risk of airway trauma (OD > ITD) was limited to 8.1% (36/446) in Group DM and 11.9% (16/134) in Group B (Supplementary Table 1).

### 3.5 Comparative predictive performance

For Group DM, the regression tree demonstrated strong predictive performance in both the training and testing datasets. The model explained a high proportion of variance in ETT size and maintained consistently low error across evaluation metrics. Importantly, performance remained stable when applied to the independent testing dataset, indicating good generalizability and minimal evidence of overfitting. Overall, the results suggest that the model provides reliable and robust predictions for Group DM (Table 1). In contrast, the predictions for Group B showed reduced generalizability. Although training datasets performance was satisfactory and explained a substantial proportion of the variance with relatively low prediction error, testing datasets error increased: the mean absolute error (MAE) reached 0.524 mm and marginally exceeded the predefined 0.5 mm clinical tolerance threshold (Table 1).

**Table 1:** Predictive performance of the decision tree models for endotracheal tube size selection in the training and independent test datasets of dolichocephalic and mesocephalic dogs combined as the non-brachycephalic Group DM and brachycephalic dogs as Group B.

Dataset	Group DM						Group B					
	*n*	*R* ^2^	MAE	MAPE	MASE	RMSE	*n*	*R* ^2^	MAE	MAPE	MASE	RMSE
Train	312	0.868	0.519	8.41	0.319	0.699	93	0.772	0.296	6.29	0.389	0.449
Test	134	0.861	0.507	8.15	0.308	0.723	41	0.265	0.524	11.7	0.747	0.694

Model performance was evaluated using the coefficient of determination (*R*^2^), mean absolute error [MAE, (mm)], mean absolute percentage error [MAPE, (%)], mean absolute scaled error (MASE), and root mean squared error (RMSE, mm). The sample size in each dataset is indicated by *n*.

To provide a clinically interpretable assessment of model performance, the predicted ETT sizes were compared with the clinician-selected tube sizes, with discrepancies categorized as overprediction (> 0.5 mm larger than actual), underprediction (> 0.5 mm smaller than actual), and acceptable accuracy (within ±0.5 mm of actual size), as summarized in Supplementary Tables 2, 3. For Group DM, the acceptable accuracy, overprediction, and underprediction rates in the training set (*n* = 312) were 57.69% (180/312), 16.99% (53/312), and 25.32% (79/312), respectively, while in the testing set (*n* = 134), these corresponded to 56.72% (76/134), 16.42% (22/134), and 26.87% (36/134), respectively (Supplementary Table 2). Similarly, for Group B, the acceptable accuracy, overprediction, and underprediction rates in the training set (*n* = 93) were 87.10% (81/93), 4.30% (4/93), and 8.60% (8/93), respectively, compared with 75.61% (31/41), 19.51% (8/41), and 4.88% (2/41) in the testing datasets (*n* = 41), respectively (Supplementary Table 3).

## 4 Discussion

Our decision tree models incorporating BW and ITD offer practical guidelines to help novice anesthetists select the ideal ETT size that minimizes airway trauma and optimizes ventilation by reducing resistance, as described by the Hagen-Poiseuille equation. Gas flow resistance is highly diameter-sensitive: halving tube radius increases resistance 16-fold (27), while each 1 mm decrease in ETT ID increases work of breathing by 34%−154% and airway resistance by 25%−100% (9). To develop specialized guidelines for brachycephalic dogs, which often have narrower tracheas, dogs were categorized into Group DM and Group B.

Based on data from the decision tree models in Figures 4, 5, possible predictor variables of breed, BW, age, sex, and ITD were selected. Previous studies in dogs have established strong correlations between ETT size and measurements such as BW and body size determined in right lateral recumbency and measured as the distance between the *margo dorsalis* of the scapula and the tip of the fourth *phalanx distalis* of the left forelimb (8–10). In human pediatrics, variables such as body length, BW, and age are also routinely used as predictors for ETT size (28–30).

The BW was a key predictor in our decision tree model but frequently led to an overestimation of ETT size in overweight or obese patients. This is similar to humans, in which obesity does not correlate with increased airway dimensions (31). Dogs with a high body condition score (BCS) are at an increased risk of tracheal collapse, particularly small breeds (32), which can cause luminal narrowing and result in a smaller ITD than expected for a dog of that weight. Consequently, selecting an ETT based solely on BW in high-BCS dogs risks overestimation.

The BW correlates with both ITD (8) and outer tracheal diameter (OTD) (9). Our analysis identified ITD as the second most important factor predicting ETT size, aligning with previous research in Beagles suggesting that optimal ETT size corresponds to 70% of the radiographic ITD (15). A caveat is that chronic respiratory disease (33) and dynamic dorsal membrane laxity can cause or mimic tracheal narrowing, underestimating ITD and potentially resulting in the selection of an undersized ETT.

Sex had a negligible effect in our population of dogs, which is in agreement with a previous study (21). This contrasts with humans, where sex often plays a significant role in ITD and as a predictor of ETT ID (34–36); in humans, sex influences height, which is a primary determinant of tracheal diameter (37). However, height differences between male and female dogs within the same breed are often minimal. Therefore, sex is a less significant factor compared to BW and ITD.

Finally, regarding age, a previous study indicates a poor correlation with both ITD and OTD (9). Certainly, age was not a significant predictor of ETT size in Group DM in our study, although it did have a slight influence in Group B. This contrasts with human pediatrics, where age-based formulas originating from Cole's formula and further refined into various age-based equations have long been used to rapidly estimate appropriate ETT sizes before emergency airway intervention (30, 38).

The prioritization of ETT ID is critical for ventilation mechanics and complies with standards recommended by the American society for testing and materials (ASTM) (39). The maximum OD is dictated by anatomical constraints, particularly at narrow points of the respiratory tract such as the cricoid cartilage and tracheal rings, because the ETT must first pass through the space between the arytenoid cartilages; selecting an ETT with an excessive OD can result in mucosal trauma, leading to complications such as post-extubation edema, subglottic inflammation and long-term stenosis, laryngeal tears, laryngeal scars and laryngoceles (27, 39). However, the tube wall thickness can vary by 2–4 mm depending on the manufacturer (39). Reliance on standard ID sizing, without accounting for manufacturer-specific OD variations therefore influences the precise anatomical fit. While the decision tree in our study provides a primary estimate for the predicted ID, it is critical to recognize that the OD is a decisive factor in airway compatibility. Consequently, it is recommended to prepare two additional tubes — one size smaller and one size larger than the primary estimate (±0.5 mm ID) — allowing the anesthetist to adjust the size during intubation.

This study has four main limitations:

1) In the absence of post-intubation radiography and cuff pressure manometry or minimal occlusive volume techniques, we could not definitively confirm optimal tracheal fit. Although effective leak-free ventilation was achieved using the ‘hiss test', unmonitored inflation risks unrecognized over- or under-inflation. Using our largest 10 mm (ID) tube in dogs >33 kg, a size typically insufficient for dogs of this size, may have required excessive cuff pressures to seal, risking tracheal capillary ischemia and mucosal damage (40).
2) Relying on retrospective data from multiple anesthesiologists at a single teaching hospital introduced inter-operator variability and single-center preference, which may lead to differences in predicted sizes compared to global standards. However, clinician choices yielded successful outcomes without gas leakage or post-extubation airway trauma.
3) For Group B, the notable decline in the coefficient of determination (*R*^2^) along with an MAE of 0.524 mm, which exceeds the 0.5 mm clinical threshold, indicates model fluctuation and limited generalizability, likely driven by a limited sample size and high anatomical variability, such as tracheal hypoplasia. Nevertheless, the training phase demonstrated sufficient performance to support the model's foundational utility. Despite the sample size constraints, decision trees were chosen over conventional statistical methods due to their superior capability to capture complex, non-linear interactions among clinical variables (including age, sex, BW, and ITD) and to guide patient-specific decision making.
4) All ITD measurements were performed by a single investigator and therefore this study assessed only intra-observer repeatability. Inter-observer reliability was not evaluated, and the reproducibility of the measurements across different operators remains to be determined.

Hence, future research using larger multi-institutional datasets is essential to eliminate single-center tendencies and refine predictive algorithms for broader clinical use. Future models should further evaluate age for brachycephalic breeds and sex for dolichocephalic and mesocephalic breeds, while incorporating multi-point tracheal measurements and BCS derived ideal body weight (IBW). Additionally, assessing inter-observer agreement will ensure measurement reliability. To fully evaluate the clinical integrity of the decision tree in spontaneously breathing anesthetized animals, assessing airway resistance, work of breathing, and ventilatory parameters via arterial blood gas analysis is warranted (41), alongside post-intubation imaging, precise cuff metrics, and long-term monitoring to track complications and establish definitive clinical guidelines.

In conclusion, our study demonstrates that BW and ITD are significant determinants for predicting the appropriate ID of ETTs in dogs. In accordance with standard clinical practice, practitioners should prepare the predicted ETT size along with one size larger and one size smaller, facilitating the selection of the most secure and appropriate fit for the individual dog's trachea. Ultimately, the study's main strength lies in its novel decision tree for ETT sizing across diverse dog breeds, especially brachycephalic dogs, offering practical guidance and confidence for novice anesthetists.

## Data Availability

The original contributions presented in the study are included in the article/Supplementary material, further inquiries can be directed to the corresponding author.

## References

[1] Briganti A Portela DA Barsotti G Romano M Breghi G. Evaluation of the endotracheal tube cuff pressure resulting from four different methods of inflation in dogs. Vet Anaesth Analg. (2012) 39:488–94. doi: 10.1111/j.1467-2995.2012.00719.x 22642369

[2] Sahiner Y. Indications for Endotracheal Intubation. In: Erbay RH, editor *Tracheal Intubation* London: IntechOpen. (2018).

[3] Brown C. Endotracheal intubation in the dog. Lab Anim. (2007) 36:23–4. doi: 10.1038/laban0207-23 17245385

[4] Yasny JS White J. Environmental implications of anesthetic gases. Anesth Prog. (2012) 59:154–8. doi: 10.2344/0003-3006-59.4.154 23241038 PMC3522493

[5] Kumar P Lehucher-Michel MP Loundou A Bregeon F Martin F Bouhadfane M. An increased risk of spontaneous abortion, congenital anomalies, and stillbirth among women occupationally exposed to anaesthetic gases: a meta-analysis of observational studies. Int Arch Occup Environ Health. (2025) 98:913–28. doi: 10.1007/s00420-025-02182-3 41247490

[6] Kiani F Jorfi S Soltani F Ghanbari S Rezaee R Mohammadi MJ. Exposure to anesthetic gases in the operating rooms and assessment of non-carcinogenic risk among health care workers. Toxicol Rep. (2023) 11:1–8. doi: 10.1016/j.toxrep.2023.06.007 37359992 PMC10285040

[7] Silva MAP Carvalho LIM Destro MV Braz LG Braz MG. From indoors to outdoors: impact of waste anesthetic gases on occupationally exposed professionals and related environmental hazards – a narrative review and update. Environ Toxicol Pharmacol. (2025) 113:104624. doi: 10.1016/j.etap.2024.104624 39732353

[8] Haider G Lorinson K Lorinson D Auer U. Development of a clinical tool to aid endotracheal tube size selection in dogs. Vet Rec. (2020) 186:157. doi: 10.1136/vr.105065 31530720

[9] Lish J Ko JC Payton ME. Evaluation of two methods of endotracheal tube selection in dogs. J Am Anim Hosp Assoc. (2008) 44:236–42. doi: 10.5326/0440236 18762559

[10] Avki S Yigitarslan K Ozgel O. Comparison of airway size with some phenotypic parameters in Dalmatian puppies: a practical method to estimate endotracheal tube size. Vet Anaesth Analg. (2006) 33:24–7. doi: 10.1111/j.1467-2995.2005.00200.x 16412129

[11] Tamura J Oyama N Matsumoto S Owaki R Hosoya K Okumura M. Unrecognized difficult airway management during anesthesia in two brachycephalic dogs with narrow cricoid cartilage. J Vet Med Sci. (2021) 83:234–40. doi: 10.1292/jvms.20-0446 33390360 PMC7972890

[12] Packer RM Tivers MS. Strategies for the management and prevention of conformation-related respiratory disorders in brachycephalic dogs. Vet Med. (2015) 6:219–32. doi: 10.2147/VMRR.S60475 30101109 PMC6067768

[13] Ševčíková MK Ševčík K Figurová M Agyagosová-Valenčáková A Hluchý M Hornáková L . Retrospective analysis of tracheal hypoplasia in brachycephalic and non-brachycephalic dogs: Inter- and intra-observer agreement of measurements. Acta Vet Hung. (2022). doi: 10.1556/004.2022.00018 36057869

[14] Grubb T Sager J Gaynor JS Montgomery E Parker JA Shafford H . 2020 AAHA Anesthesia and Monitoring Guidelines for Dogs and Cats. J Am Anim Hosp Assoc. (2020) 56:59–82. doi: 10.5326/JAAHA-MS-7055 32078360

[15] Shin CW Son WG Jang M Kim H Han H Cha J . Selection of appropriate endotracheal tube size using thoracic radiography in Beagle dogs. Vet. Anaesth. Analg. (2018) 45:13–21. doi: 10.1016/j.vaa.2017.10.002 29246712

[16] Nawaz M Ngo V Hari A. Animal classification in wildlife through images using statistical methods and decision tree (Master's thesis). Rhine–Waal University of Applied Sciences (2015).

[17] Averós X Lavín JL Estevez I. The potential of decision trees as a tool to simplify broiler chicken welfare assessments. Sci Rep. (2024) 14:22943. doi: 10.1038/s41598-024-74260-4 39358453 PMC11447258

[18] Aerts J Kolenda M Piwczyński D Sitkowska B Önder H. Forecasting milking efficiency of dairy cows milked in an automatic milking system using the decision tree technique. Animals. (2022) 12:1040. doi: 10.3390/ani12081040 35454286 PMC9024698

[19] Engel-Manchado J Montoya-Alonso JA Doménech L Monge-Utrilla O Reina-Doreste Y Matos JI . Machine learning techniques for canine myxomatous mitral valve disease classification: integrating anamnesis, quality of life survey, and physical examination. Vet Sci. (2024) 11:118. doi: 10.3390/vetsci11030118 38535852 PMC10974186

[20] Tamburis O Masciari E Fatone G. Development of a decision tree model to improve case detection via information extraction from veterinary Electronic Medical Records. In: Greco S Lenzerini M Masciari E Tagarelli A, editor. SEBD 2021: Proceedings of the 29th Italian Symposium on Advanced Database Systems. Pizzo Calabro (VV) (2021). p. 105–15.

[21] Khiao-In M Kananub S Sritrakul T Thamsatit P Theerawatanasirikul S Srisowanna N. Predicting dog tracheal diameter and length: a tool for improved intubation. J Adv Vet Anim Res. (2024) 11:323–9. doi: 10.5455/javar.2024.k780 39101101 PMC11296161

[22] Ekenstedt KJ Crosse KR Risselada M. Canine brachycephaly: anatomy, pathology, genetics and welfare. J Comp Pathol. (2020) 176:109–15. doi: 10.1016/j.jcpa.2020.02.008 32359622 PMC7380493

[23] Tomlinson F Liu NC Sargan DR Ladlow JF A. cross-sectional study into the prevalence and conformational risk factors of BOAS across fourteen brachycephalic dog breeds. PLoS ONE. (2026) 21:e0340604. doi: 10.1371/journal.pone.0340604 41706647 PMC12915975

[24] Kuhn M. Caret: Classification and Regression Training (2024). Available online at: https://CRAN.R-project.org/package=caret (Accessed 2025).

[25] Boehmke B Greenwell B. Hands-On Machine Learning with R. New York, NY: Chapman and Hall/CRC (2019). doi: 10.1201/9780367816377

[26] Amirhosseini MH Yadav V Serpell J Pettigrew P Kain P. An artificial intelligence approach to predicting personality types in dogs. Sci Rep. (2024) 14. doi: 10.1038/s41598-024-52920-9 38286813 PMC10825194

[27] Farrow S Farrow C Soni N. Size matters: choosing the right tracheal tube. Anaesthesia. (2012) 67:815–9. doi: 10.1111/j.1365-2044.2012.07250.x 22775368

[28] Eipe N Barrowman N Writer H Doherty D A. weight-based formula for tracheal tube size in children. Paediatr Anaesth. (2009) 19:343–8. doi: 10.1111/j.1460-9592.2008.02916.x 19178655

[29] Luten RC Wears RL Broselow J Zaritsky A Barnett TM Lee T . Length-based endotracheal tube and emergency equipment in pediatrics. Ann Emerg Med. (1992) 21:900–4. doi: 10.1016/S0196-0644(05)82924-5 1497153

[30] King BR Baker MD Braitman LE Seidl-Friedman J Schreiner MS. Endotracheal tube selection in children: a comparison of four methods. Ann Emerg Med. (1993) 22:530–4. doi: 10.1016/S0196-0644(05)81937-7 8442540

[31] D'Anza B Knight J Greene JS. Do obese patients need larger endotracheal tubes? OHNS. (2013) 149:P47–8. doi: 10.1177/0194599813495815a46

[32] Kim MR Kim SH Ryu MO Youn HY Choi JH Seo KW. A retrospective study of tracheal collapse in small-breed dogs: 110 cases (2022–2024). Front Vet Sci. (2024) 11 doi: 10.3389/fvets.2024.1448249 39205804 PMC11349713

[33] Della Maggiore A. An update on tracheal and airway collapse in dogs. Vet Clin North Am Small Anim Pract. (2020) 50:419–30. doi: 10.1016/j.cvsm.2019.11.003 31864678

[34] Karmakar A Pate MB Solowski NL Postma GN Weinberger PM. Tracheal size variability is associated with sex: implications for endotracheal tube selection. Ann Otol Rhinol Laryngol. (2015) 124:132–6. doi: 10.1177/0003489414549154 25305266

[35] Tai A Corke C Joynt GM Griffith J Lunn D Tong P. A comparative study of tracheal diameter in Caucasian and Chinese patients. Anaesth Intensive Care. (2016) 44:719–23. doi: 10.1177/0310057X1604400603 27832558

[36] Aljathlany Y Aljasser A Alhelali A Bukhari M Almohizea M Khan A . Proposing an endotracheal tube selection tool based on multivariate analysis of airway imaging. Ear Nose Throat J. (2021) 100:629S−35S. doi: 10.1177/0145561319900390 31914813

[37] Coordes A Rademacher G Knopke S Todt I Ernst A Estel B . Selection and placement of oral ventilation tubes based on tracheal morphometry. Laryngoscope. (2011) 121:1225–30. doi: 10.1002/lary.21752 21557233

[38] Manimalethu R Krishna S Shafy SZ Hakim M Tobias JD. Choosing endotracheal tube size in children: which formula is best? Int J Pediatr Otorhinolaryngol. (2020) 134:110016. doi: 10.1016/j.ijporl.2020.110016 32247219

[39] Gupta B Gupta L. Significance of the outer diameter of an endotracheal tube: a lesser-known parameter. Korean J Anesthesiol. (2019) 72:72–3. doi: 10.4097/kja.d.18.00056 29843505 PMC6369347

[40] Hung WC Ko JC Weil AB Weng HY. Evaluation of Endotracheal Tube Cuff Pressure and the Use of Three Cuff Inflation Syringe Devices in Dogs. Front Vet Sci. (2020) 7:39. doi: 10.3389/fvets.2020.00039 32118062 PMC7015870

[41] Wagner PD. The physiological basis of pulmonary gas exchange: implications for clinical interpretation of arterial blood gases. Eur Respir J. (2015) 45:227–43. doi: 10.1183/09031936.00039214 25323225

