# Supplementary material for: Decision tree models to guide endotracheal tube selection in dogs
Source: Front Vet Sci. 2026 Oct 21;13:1894775. doi: 10.3389/fvets.2026.1894775 (PMC13732578; doi:10.3389/fvets.2026.1894775)
Supplement: Supplementary file 1 [file Data_Sheet_1.PDF]

R code for decision tree

```
library(dplyr)
library(tidyr)
library(rpart)
library(rpart.plot)
library(caret)
library(ggplot2)

set.seed(1234)

target_type <- "Short"

D446 <- read_csv("D446.csv")

df <- D446 %>%
  filter(Type == target_type) %>%
  select(ET, ITD, BW, Age_months, Sex) %>%
  mutate(
    ET = as.numeric(ET),
    ITD = as.numeric(ITD),
    BW = as.numeric(BW),
    Age_months = as.numeric(Age_months),
    Sex = factor(Sex)
  ) %>%
  drop_na()

str(df)
table(df$Sex)

num_vars <- c("ITD", "BW", "Age_months")

num_screen <- lapply(num_vars, function(v) {
  ct <- cor.test(
    df[[v]],
    df$ET,
    method = "pearson"
  )

  tibble(
    variable = v,
    type = "numeric",
    statistic = unname(ct$estimate),
    p_value = ct$p.value
```

```
  )
}) %>%
  bind_rows()

cat_vars <- c("Sex")

cat_screen <- lapply(cat_vars, function(v) {
  kw <- kruskal.test(
    df$ET,
    df[[v]]
  )

  tibble(
    variable = v,
    type = "categorical",
    statistic = unname(kw$statistic),
    p_value = kw$p.value
  )
}) %>%
  bind_rows()

screen_table <- bind_rows(
  num_screen,
  cat_screen
) %>%
  mutate(
    pass_0.05 = p_value < 0.05
  ) %>%
  arrange(p_value)

print(screen_table)

keep_num <- screen_table %>%
  filter(
    type == "numeric",
    pass_0.05
  ) %>%
  pull(variable)

keep_cat <- screen_table %>%
  filter(
    type == "categorical",
    pass_0.05
  ) %>%
```

```r
  pull(variable)

if (length(keep_num) > 1) {
  correlation_matrix <- cor(
    df[, keep_num, drop = FALSE],
    use = "pairwise.complete.obs"
  )

  drop_variables <- findCorrelation(
    correlation_matrix,
    cutoff = 0.90,
    names = TRUE,
    exact = TRUE
  )

  keep_num2 <- setdiff(
    keep_num,
    drop_variables
  )
} else {
  keep_num2 <- keep_num
}

print(keep_num2)

rfe_selected <- keep_num2

if (length(keep_num2) >= 2) {
  rfe_control <- rfeControl(
    functions = lmFuncs,
    method = "repeatedcv",
    number = 10,
    repeats = 5,
    verbose = FALSE
  )

  rfe_fit <- rfe(
    x = df[, keep_num2, drop = FALSE],
    y = df$ET,
    sizes = seq_along(keep_num2),
    rfeControl = rfe_control,
    metric = "RMSE"
  )
```

```
  rfe_selected <- predictors(rfe_fit)

  print(rfe_fit)
}

print(rfe_selected)

final_vars <- union(
  rfe_selected,
  keep_cat
)

if (length(final_vars) == 0) {
  stop("No predictor variables passed the selection process.")
}

print(final_vars)

train_index <- createDataPartition(
  df$ET,
  p = 0.70,
  list = FALSE
)

train <- df[train_index, ]
test <- df[-train_index, ]

cat("Training observations:", nrow(train), "\n")
cat("Testing observations:", nrow(test), "\n")

model_formula <- reformulate(
  final_vars,
  response = "ET"
)

fit0 <- rpart(
  formula = model_formula,
  data = train,
  method = "anova",
  xval = 10,
  control = rpart.control(
    cp = 0.001,
    minsplit = 15,
    minbucket = 7,
```

```
    maxdepth = 30
  )
)

print(fit0)
print(fit0$cptable)

best_cp <- fit0$cptable[
  which.min(fit0$cptable[, "xerror"]),
  "CP"
]

fit <- prune(
  fit0,
  cp = best_cp
)

print(fit)
print(fit$cptable)

pred_train <- as.numeric(
  predict(
    fit,
    newdata = train
  )
)

pred_test <- as.numeric(
  predict(
    fit,
    newdata = test
  )
)

rmse <- function(actual, predicted) {
  sqrt(
    mean(
      (actual - predicted)^2
    )
  )
}

mae <- function(actual, predicted) {
  mean(
```

```
    abs(
      actual - predicted
    )
  )
}

r2 <- function(actual, predicted) {
  1 -
    sum((actual - predicted)^2) /
    sum((actual - mean(actual))^2)
}

mape <- function(actual, predicted) {
  mean(
    abs(
      (actual - predicted) / actual
    )
  ) * 100
}

base_const <- mean(train$ET)

baseline_train_mae <- mae(
  train$ET,
  rep(base_const, nrow(train))
)

baseline_test_mae <- mae(
  test$ET,
  rep(base_const, nrow(test))
)

metrics <- bind_rows(
  tibble(
    Dataset = "Training",
    n = nrow(train),
    R2 = r2(train$ET, pred_train),
    MAE = mae(train$ET, pred_train),
    MAPE = mape(train$ET, pred_train),
    MASE = mae(train$ET, pred_train) /
      baseline_train_mae,
    RMSE = rmse(train$ET, pred_train)
  ),
  tibble(
```

```
    Dataset = "Testing",
    n = nrow(test),
    R2 = r2(test$ET, pred_test),
    MAE = mae(test$ET, pred_test),
    MAPE = mape(test$ET, pred_test),
    MASE = mae(test$ET, pred_test) /
      baseline_test_mae,
    RMSE = rmse(test$ET, pred_test)
  )
)

print(metrics)

pred_baseline_test <- rep(
  base_const,
  nrow(test)
)

baseline_metrics <- tibble(
  Dataset = "Baseline",
  n = nrow(test),
  R2 = r2(test$ET, pred_baseline_test),
  MAE = mae(test$ET, pred_baseline_test),
  MAPE = mape(test$ET, pred_baseline_test),
  MASE = 1,
  RMSE = rmse(test$ET, pred_baseline_test)
)

print(baseline_metrics)

final_metrics <- bind_rows(
  metrics,
  baseline_metrics
) %>%
  mutate(
    across(
      c(R2, MAE, MAPE, MASE, RMSE),
      ~ round(.x, 3)
    )
  )

print(final_metrics)

if (
```

```r
    !is.null(fit$variable.importance) &&
    length(fit$variable.importance) > 0
  ) {
    importance <- tibble(
      Variable = names(fit$variable.importance),
      Importance = as.numeric(fit$variable.importance)
    ) %>%
      mutate(
        Relative_importance =
          100 * Importance / max(Importance)
      ) %>%
      arrange(Relative_importance) %>%
      mutate(
        Variable = factor(
          Variable,
          levels = Variable
        )
      )

    print(importance)

    importance_plot <- ggplot(
      importance,
      aes(
        x = Variable,
        y = Relative_importance
      )
    ) +
      geom_col(width = 0.6) +
      geom_text(
        aes(
          label = sprintf(
            "%.1f",
            Relative_importance
          )
        ),
        hjust = -0.1,
        size = 3.8
      ) +
      coord_flip(
        clip = "off"
      ) +
      labs(
        title = paste(
```

```
        "Decision Tree Variable Importance:",
        target_type
      ),
      x = NULL,
      y = "Relative importance (%)"
    ) +
    theme_minimal(
      base_size = 13
    ) +
    scale_y_continuous(
      expand = expansion(
        mult = c(0, 0.10)
      )
    )

  print(importance_plot)
}

rpart.plot(
  fit,
  type = 2,
  extra = 101,
  under = TRUE,
  fallen.leaves = TRUE,
  branch = 0.4,
  faclen = 0,
  digits = 3,
  roundint = FALSE,
  box.palette = "RdBu",
  tweak = 1.1,
  main = target_type
)

png(
  filename = paste0(
    "ET_tree_",
    target_type,
    ".png"
  ),
  width = 2800,
  height = 1400,
  res = 220
)
```

```
rpart.plot(
  fit,
  type = 2,
  extra = 101,
  under = TRUE,
  fallen.leaves = TRUE,
  branch = 0.4,
  faclen = 0,
  digits = 3,
  roundint = FALSE,
  box.palette = "RdBu",
  tweak = 1.1,
  main = target_type
)

dev.off()

rpart.rules(
  fit,
  cover = TRUE,
  roundint = FALSE
)










library(randomForest)
mtry_candidates <- seq_len(length(final_vars))

rf_tuning <- lapply(mtry_candidates, function(m) {
  set.seed(1234)

  rf_temp <- randomForest(
    formula = model_formula,
    data = train,
    ntree = 500,
    mtry = m,
    nodesize = 5,
```

```r
    importance = TRUE
  )

  tibble(
    mtry = m,
    OOB_MSE = tail(rf_temp$mse, 1),
    OOB_RMSE = sqrt(tail(rf_temp$mse, 1)),
    OOB_R2 = tail(rf_temp$rsq, 1)
  )
}) %>%
  bind_rows()

print(rf_tuning)

best_mtry <- rf_tuning$mtry[
  which.min(rf_tuning$OOB_RMSE)
]

cat("Selected mtry:", best_mtry, "\n")

set.seed(1234)

rf_fit <- randomForest(
  formula = model_formula,
  data = train,
  ntree = 1000,
  mtry = best_mtry,
  nodesize = 5,
  importance = TRUE,
  keep.forest = TRUE
)

print(rf_fit)

rf_pred_train <- as.numeric(
  predict(
    rf_fit,
    newdata = train
  )
)

rf_pred_test <- as.numeric(
  predict(
    rf_fit,
```

```
    newdata = test
  )
)

rf_metrics <- bind_rows(
  tibble(
    Model = "Random Forest",
    Dataset = "Training",
    n = nrow(train),
    R2 = r2(train$ET, rf_pred_train),
    MAE = mae(train$ET, rf_pred_train),
    MAPE = mape(train$ET, rf_pred_train),
    MASE = mae(train$ET, rf_pred_train) /
      baseline_train_mae,
    RMSE = rmse(train$ET, rf_pred_train)
  ),
  tibble(
    Model = "Random Forest",
    Dataset = "Testing",
    n = nrow(test),
    R2 = r2(test$ET, rf_pred_test),
    MAE = mae(test$ET, rf_pred_test),
    MAPE = mape(test$ET, rf_pred_test),
    MASE = mae(test$ET, rf_pred_test) /
      baseline_test_mae,
    RMSE = rmse(test$ET, rf_pred_test)
  )
) %>%
  mutate(
    across(
      c(R2, MAE, MAPE, MASE, RMSE),
      ~ round(.x, 3)
    )
  )

print(rf_metrics)

dt_metrics <- metrics %>%
  mutate(
    Model = "Decision Tree"
  ) %>%
  select(
    Model,
    Dataset,
```

```
    n,
    R2,
    MAE,
    MAPE,
    MASE,
    RMSE
  )

model_comparison <- bind_rows(
  dt_metrics,
  rf_metrics
) %>%
  mutate(
    across(
      c(R2, MAE, MAPE, MASE, RMSE),
      ~ round(.x, 3)
    )
  ) %>%
  arrange(
    Dataset,
    Model
  )

print(model_comparison)

rf_oob_results <- tibble(
  Number_of_trees = rf_fit$ntree,
  Selected_mtry = rf_fit$mtry,
  Final_OOB_MSE = tail(rf_fit$mse, 1),
  Final_OOB_RMSE = sqrt(tail(rf_fit$mse, 1)),
  Final_OOB_R2 = tail(rf_fit$rsq, 1)
) %>%
  mutate(
    across(
      where(is.numeric),
      ~ round(.x, 4)
    )
  )

print(rf_oob_results)

rf_importance_raw <- randomForest::importance(
  rf_fit,
  scale = FALSE
```

```
)

rf_importance <- as.data.frame(
  rf_importance_raw
) %>%
  tibble::rownames_to_column(
    var = "Variable"
  ) %>%
  transmute(
    Variable,
    Permutation_importance = `%IncMSE`,
    Increase_node_purity = IncNodePurity,
    Positive_importance = pmax(
      Permutation_importance,
      0
    )
  )

if (
  max(
    rf_importance$Positive_importance,
    na.rm = TRUE
  ) <= 0
) {
  stop(
    "All permutation importance values are zero or negative."
  )
}

rf_importance <- rf_importance %>%
  mutate(
    Relative_importance_percent =
      100 *
      Positive_importance /
      max(
        Positive_importance,
        na.rm = TRUE
      )
  ) %>%
  arrange(
    desc(Relative_importance_percent)
  ) %>%
  mutate(
    Importance_rank = row_number(),
```

```
    Permutation_importance =
      round(Permutation_importance, 4),
    Increase_node_purity =
      round(Increase_node_purity, 4),
    Relative_importance_percent =
      round(Relative_importance_percent, 2)
  ) %>%
  select(
    Importance_rank,
    Variable,
    Permutation_importance,
    Relative_importance_percent,
    Increase_node_purity
  )

print(rf_importance)

rf_importance_plot_data <- rf_importance %>%
  arrange(
    Relative_importance_percent
  ) %>%
  mutate(
    Variable = factor(
      Variable,
      levels = Variable
    )
  )

rf_importance_plot <- ggplot(
  rf_importance_plot_data,
  aes(
    x = Variable,
    y = Relative_importance_percent,
    fill = Variable
  )
) +
  geom_col(
    width = 0.6
  ) +
  geom_text(
    aes(
      label = paste0(
        sprintf(
          "%.1f",
```

```
          Relative_importance_percent
        ),
        "%"
      )
    ),
    hjust = -0.1,
    size = 3.8
  ) +
  coord_flip(
    clip = "off"
  ) +
  scale_fill_manual(
    values = c(
      "BW" = "#0B9D8F",
      "ITD" = "#53B6AE",
      "Age_months" = "#80C8C2",
      "Sex" = "#B2DEDA"
    )
  ) +
  scale_x_discrete(
    labels = c(
      "BW" = "Body weight (kg)",
      "ITD" = "Internal tracheal diameter (mm)",
      "Age_months" = "Age (Month)",
      "Sex" = "Sex"
    )
  ) +
  labs(
    x = NULL,
    y = "Importance Score"
  ) +
  theme_bw(
    base_size = 13
  ) +
  theme(
    plot.title = element_text(
      face = "bold"
    ),
    legend.position = "none",
    panel.grid.major.y = element_blank(),
    panel.grid.minor = element_blank(),
    plot.margin = ggplot2::margin(
      10,
      45,
```

```
      10,
      10,
      unit = "pt"
    )
  ) +
  scale_y_continuous(
    limits = c(0, 110),
    breaks = seq(
      0,
      100,
      by = 25
    ),
    expand = expansion(
      mult = c(0, 0)
    )
  )

print(rf_importance_plot)

ggsave(
  filename = paste0(
    "Random_forest_variable_importance_",
    target_type,
    ".png"
  ),
  plot = rf_importance_plot,
  width = 8,
  height = 5,
  units = "in",
  dpi = 300
)
```
