# Supplementary material for: Decision tree models to guide endotracheal tube selection in dogs
Source: Front Vet Sci. 2026 Oct 21;13:1894775. doi: 10.3389/fvets.2026.1894775 (PMC13732578; doi:10.3389/fvets.2026.1894775)
Supplement: Supplementary file 2 [file Supplementary_file_1.pdf]

Supplementary Material

Supplementary Figures

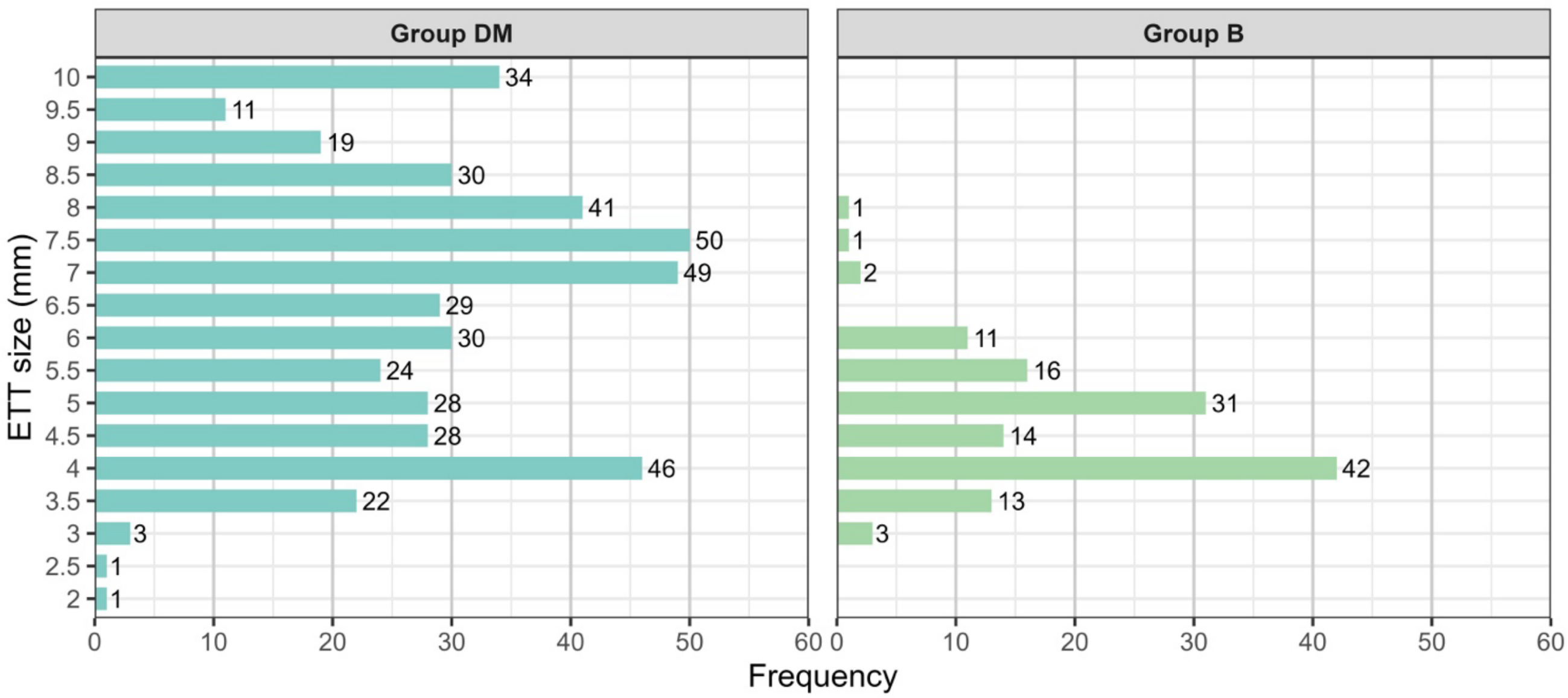

Supplementary Figure 1. Frequency distribution of endotracheal tube (ETT) sizes. Bar charts display the frequency of selected ETT sizes internal diameter (ID) used in intubated dogs, comparing dolichocephalic and mesocephalic dogs (Group DM) and brachycephalic dogs (Group B). Numerical values at the end of each bar indicate the absolute count of cases for each tube size.

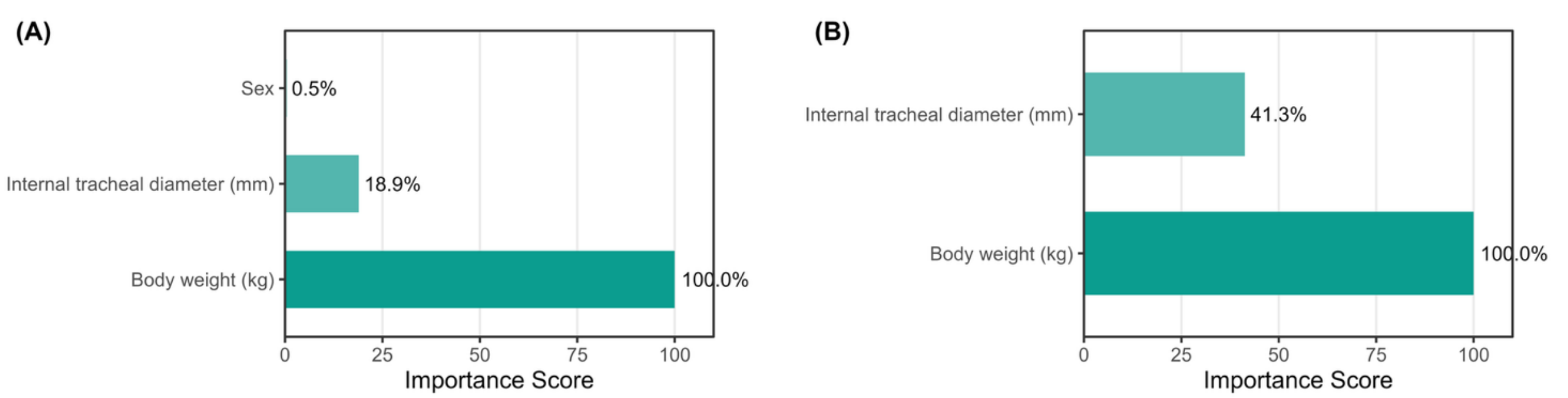

Supplementary Figure 2. Relative variable importance scores derived from random forest analysis. Predictive contribution of anatomical and demographic variables for estimating endotracheal tube size in (A) dolichocephalic and mesocephalic dogs (Group DM) and (B) brachycephalic dogs (Group B). Bar charts show the relative importance scores (%) of sex, internal tracheal diameter (ITD; millimeters (mm)) and body weight (BW; kilograms (kg)).

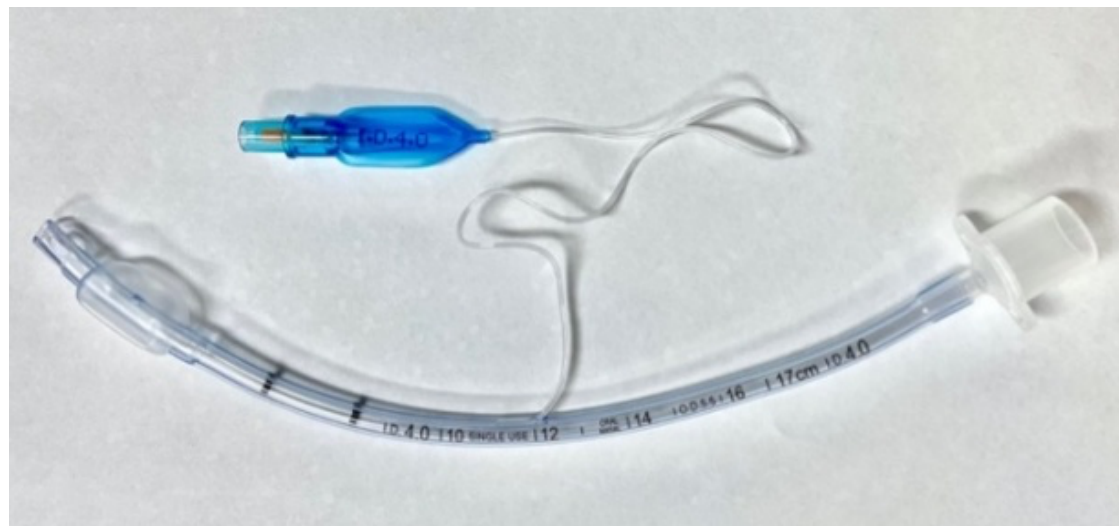

Supplementary Figure 3. Structural dimensions of a representative endotracheal tube (ETT). Photograph of ETT illustrating printed internal diameter (ID 4.0 mm).

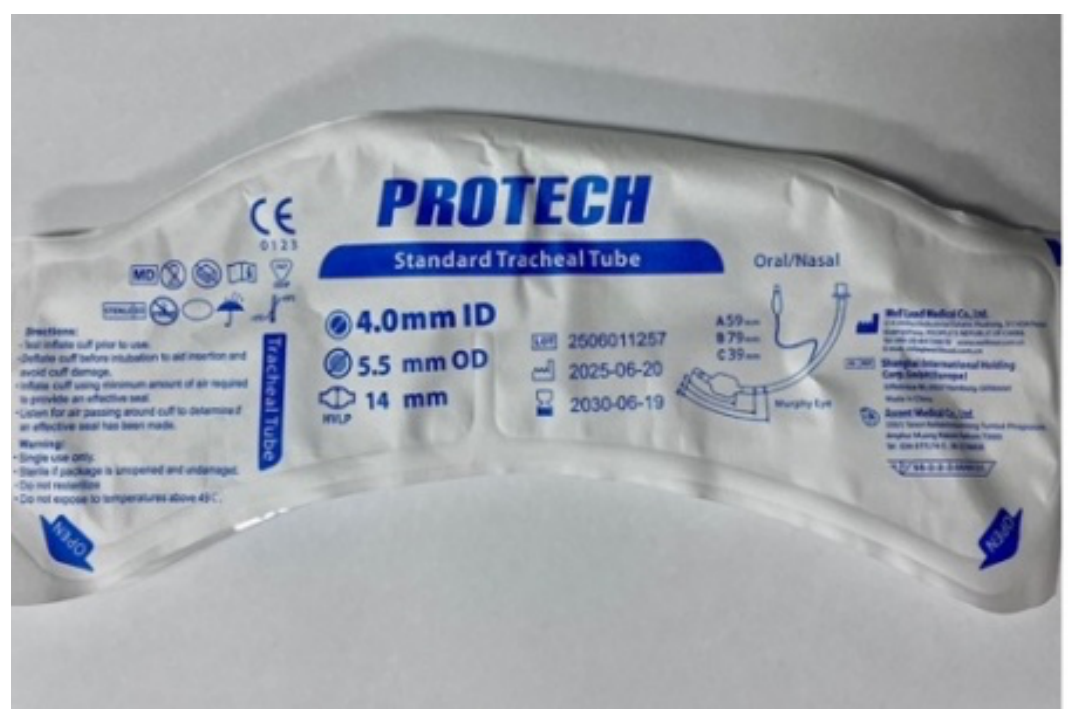

Supplementary Figure 4. Manufacturer packaging displaying endotracheal tube (ETT) dimensional specifications. Photograph of the commercial ETT sterile packaging highlighting manufacturer-labeled specifications, including internal diameter (ID 4.0 mm) and outer diameter (OD 5.5 mm).
