# Supplementary material for: Decision tree models to guide endotracheal tube selection in dogs
Source: Front Vet Sci. 2026 Oct 21;13:1894775. doi: 10.3389/fvets.2026.1894775 (PMC13732578; doi:10.3389/fvets.2026.1894775)
Supplement: Supplementary file 3 [file Supplementary_file_2.pdf]

Supplementary Material

Supplementary Tables

Supplementary Table 1. Comparison of predicted endotracheal tube (ETT) outer diameter (OD) relative to internal tracheal diameter (ITD) in dolichocephalic and mesocephalic dogs (Group DM) and brachycephalic dogs (Group B). Values are presented as n (%). OD ≤ ITD indicates instances where the predicted ETT OD did not exceed the measured ITD, suggesting a low likelihood of airway injury. OD > ITD denotes cases where the expected OD exceeded the ITD, representing a potential risk for airway trauma.

Group	Expected OD ≤ ITD	Expected OD > ITD	Total (n)
DM	410 (91.9%)	36 (8.1%)	446
B	118 (88.1%)	16 (11.9%)	134
Overall	528 (91.0%)	52 (9.0%)	580

Supplementary Table 2. Comparison between decision tree-expected and actual endotracheal tube (ETT) sizes in dolichocephalic and mesocephalic dogs (Group DM) across training and testing sets. Discrepancies between expected and actual ETT sizes are categorized as overprediction (> 0.5 mm larger than actual), underprediction (> 0.5 mm smaller than actual), and acceptable accuracy (within±0.5 mm of actual size).

ETT	Train			Test		
	Overprediction by more than 0.5 mm	Underprediction by more than 0.5 mm	Within ±0.5 mm	Overprediction by more than 0.5 mm	Underprediction by more than 0.5 mm	Within ±0.5 mm
2	1	n/a	n/a	n/a	n/a	n/a
2.5	1	n/a	n/a	n/a	n/a	n/a

3	2	n/a	n/a	1	n/a	n/a
3.5	2	n/a	14	3	n/a	3
4	3	n/a	26	2	n/a	15
4.5	6	3	10	3	4	2
5	2	4	13	n/a	1	8
5.5	4	2	8	3	1	6
6	4	4	15	n/a	3	4
6.5	8	n/a	12	3	2	4
7	11	11	13	2	3	9
7.5	3	4	27	3	1	12
8	3	6	20	1	3	8
8.5	3	12	8	1	2	4
9	n/a	8	9	n/a	1	1
9.5	n/a	4	5	n/a	2	n/a
10	n/a	21	n/a	n/a	13	n/a

Supplementary Table 3. Comparison between decision tree-expected and actual endotracheal tube (ETT) sizes in brachycephalic dogs (Group B) across training and testing sets. Discrepancies between expected and actual ETT sizes are categorized as overprediction (> 0.5 mm larger than actual), underprediction (> 0.5 mm smaller than actual), and acceptable accuracy (within ±0.5 mm of actual size).

ETT	Train			Test		
	Overprediction by more than 0.5 mm	Underprediction by more than 0.5 mm	Within ±0.5 mm	Overprediction by more than 0.5 mm	Underprediction by more than 0.5 mm	Within ±0.5 mm
3	1	n/a	n/a	2	n/a	n/a
3.5	n/a	n/a	10	n/a	n/a	3
4	1	n/a	31	1	n/a	9
4.5	n/a	n/a	11	1	n/a	2
5	1	n/a	17	2	n/a	11
5.5	1	3	6	2	1	3
6	n/a	1	6	n/a	1	3
6.5	n/a	n/a	n/a	n/a	n/a	n/a
7	n/a	2	n/a	n/a	n/a	n/a
7.5	n/a	1	n/a	n/a	n/a	n/a
8	n/a	1	n/a	n/a	n/a	n/a
